# Supplementary material for: Functional and epigenetic phenotypes of humans and mice with DNMT3A Overgrowth Syndrome
Source: Nat Commun. 2021 Jul 27;12:4549. doi: 10.1038/s41467-021-24800-7 (PMC8316576; doi:10.1038/s41467-021-24800-7)
Supplement: Supplementary file 1 — Supplementary Information [file 41467_2021_24800_MOESM1_ESM.pdf]

a

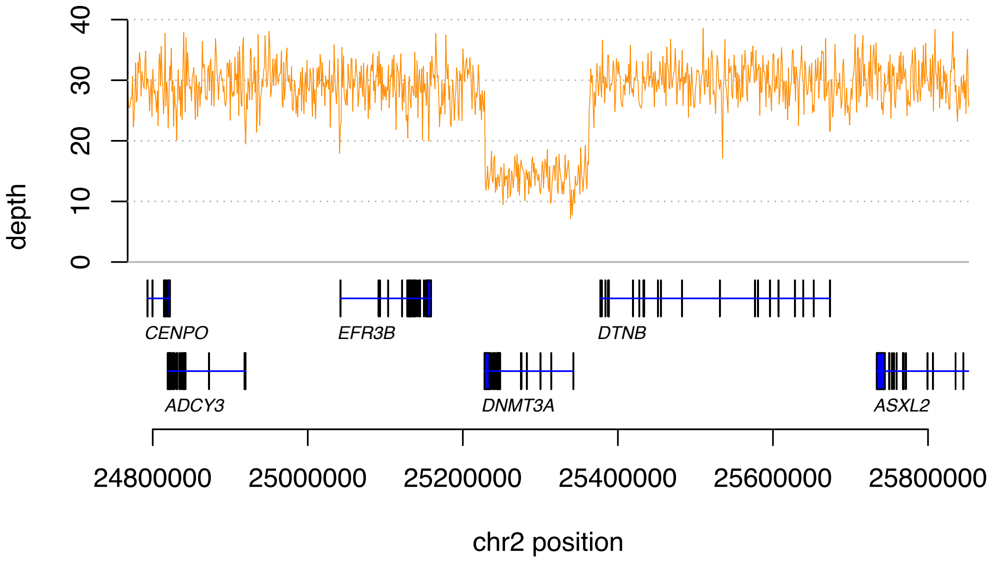

b

Category	Associated DMRs	% DMR associated#
CpG Islands (n=31,144)	564	1.81%
CpG Shores (n=56,288)	955	1.70%
CpG Shelves (n=47,308)	214	0.45%
Gene Body (n=18,951)***	1426	7.52%
Promoters (n=38,459)	923	2.4%
TSS (n=83,014)	177	0.21%
Enhancers (n=121,622)	76	0.06%

c

Category	Associated DMRs	% DMR associated#
CpG Islands (n=16,023)	207	1.29%
CpG Shores (n=30,212)	490	1.62%
CpG Shelves (n=43,429)	187	0.43%
Gene Body (n=22,026)***	1375	6.24%
Promoters (n=22,271)	677	3.04%
TSS (n=57,853)	151	0.26%
Enhancers (n=68,260)	53	0.08%

Supplementary Figure 1

Supplementary Figure 1. Structure of DNMT3A deletion in UPN518693. a) Deletion of the *DNMT3A* gene in UPN 518593. b) Summary of the number of human *DNMT3A*^{R882} DMRs within each annotated region in the genome, as well as percentage affected for each region (***p ≤ 0.001, Fisher's Exact Test). # Percentage is calculated on unique regions (i.e. if one gene body is affected by >1 DMR, it was only included once to calculate frequencies). c) Summary of the number of mouse *Dnmt3a*$^{R878H/+}$ DMRs within each annotated region in the genome, as well as percentage of affected for each region (***p≤0.001, Fisher's Exact Test). # Percentage is calculated on unique regions (i.e. if one gene body is affected by >1 DMR, it was only included once to calculate frequencies). Chr; chromosome, TSS; transcriptional start site

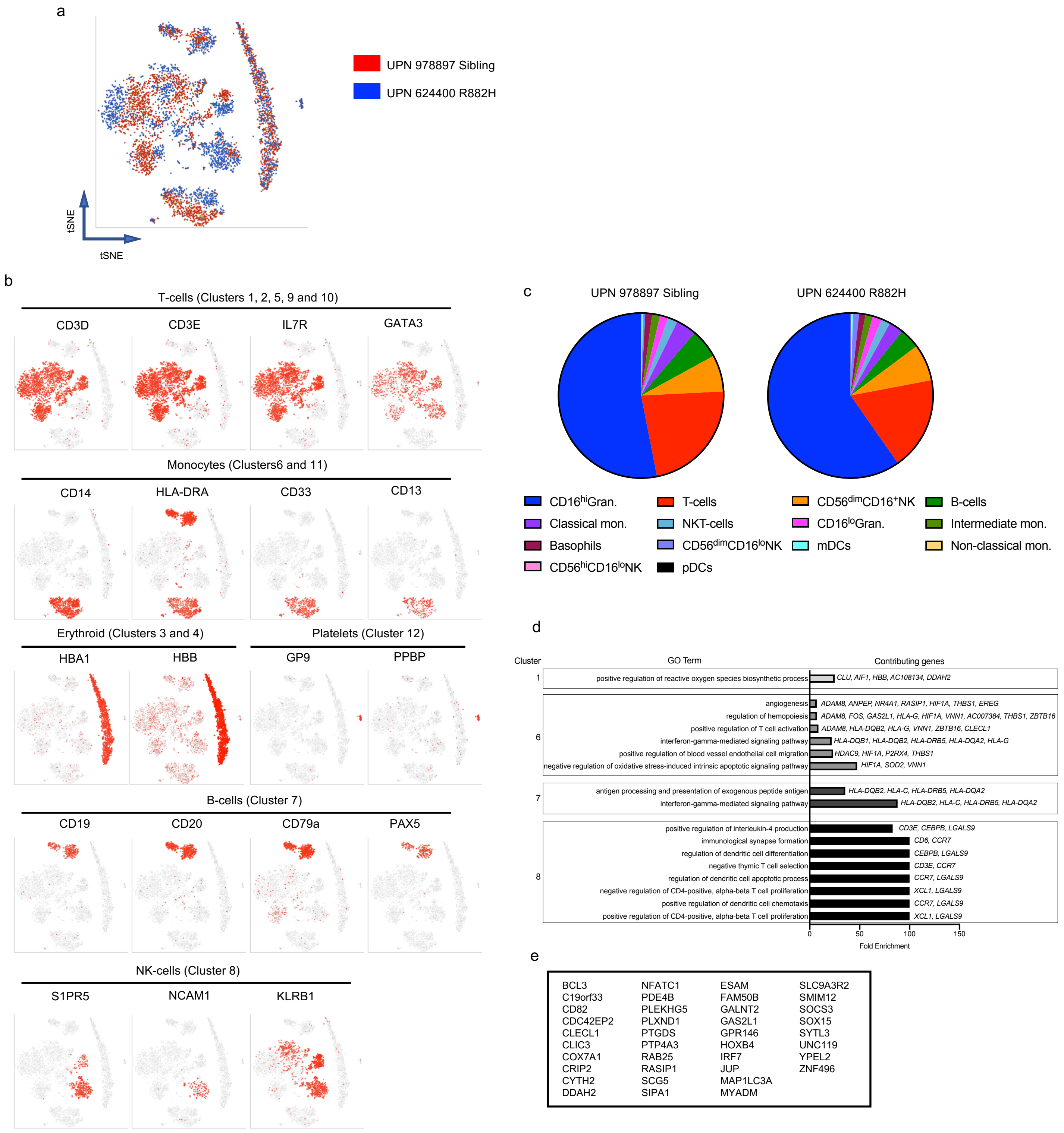

Supplementary Figure [illegible]

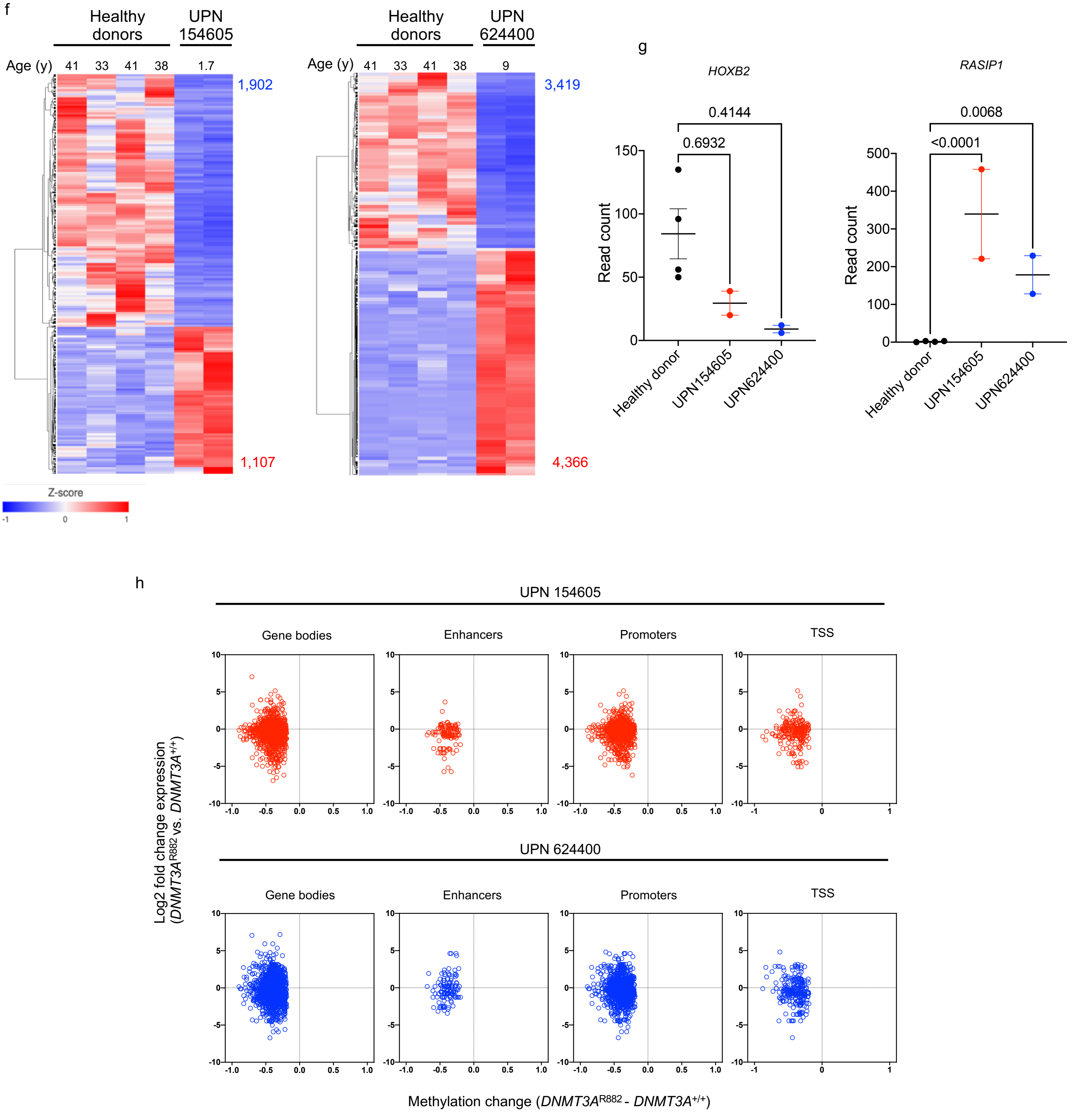

Supplementary Figure 2

Supplementary Figure 2. *Germline DNMT3A^{R882} is associated with differences in transcriptional activity in the peripheral blood* a) tSNE projection of scRNA-seq data from fresh peripheral blood derived from one DOS patient with an R882H mutation (UPN 624400) and his matched *DNMT3A$^{+/+}$* sibling control sample (UPN 978897). b) tSNE projections of lineage-defining genes, validating Toppfun identified cell populations within graph-based clusters. c) Quantification of flow cytometry data from the same samples used for scRNA-seq, confirming population alterations. Each defined population is shown as a percentage of CD45+ cells. d) Gene ontology terms for DEGs in the indicated clusters, with contributing genes listed on the right. Clusters not identified did not have associated GO terms. e) 38 genes identified as differentially expressed in *DNMT3A*^{R882} by scRNA-seq, and located within 10 kb of a DMR. f) Heatmap of normalized (Z-score) expression values for differentially expressed genes identified by bulk RNA-seq defined by comparing UPN 154605 (left) or UPN 624400 (right) to healthy donor peripheral blood. Both patients have the *DNMT3A*^{R882H} mutation. g) Expression of *HOXB2* and *RASIP1* from bulk RNA-seq of peripheral blood from UPN154605 (2 technical replicates), UPN 624400 (2 technical replicates) and the same healthy donors (n=4). P-values (determined by ANOVA) are indicated, bars represent mean ± SEM. h) Expression vs. methylation difference plots for all expressed genes between UPN 154605 (*red*) and UPN 624400 (*blue*) and unaffected donors, that are associated with a DMR. Functional locations of DMRs are defined, and plotted separately (gene bodies, enhancers, promoters, and Transcription Start Sites/TSS). GO; gene ontology, y; years.

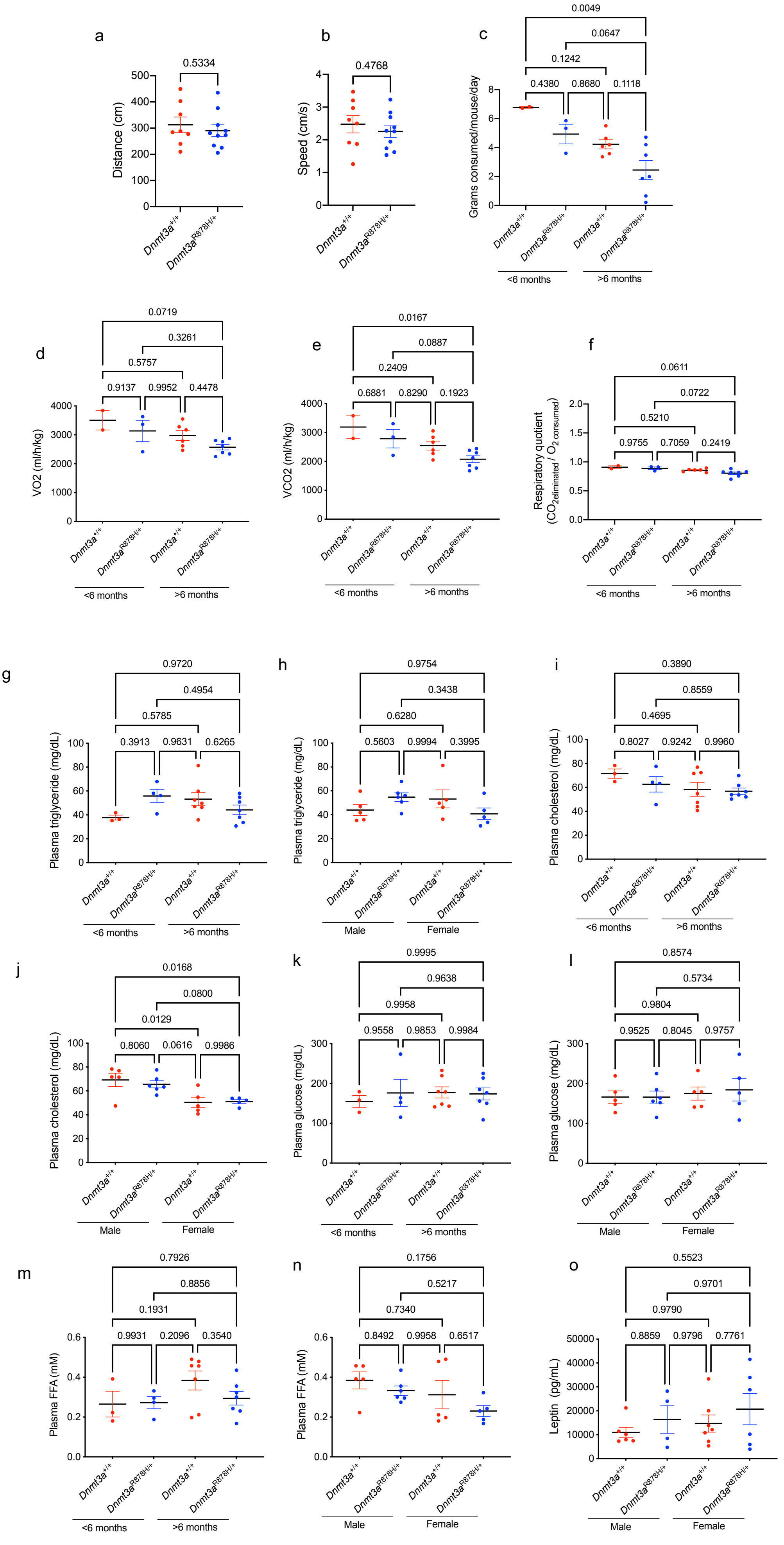

Supplementary Figure 3

Supplementary Figure 3. Germline Dnmt3a$^{R878H/+}$ mice have normal blood metabolic profiles. Metabolic cage analysis measurements over a 72-hour period of a) distance travelled b) average speed of travel, c) food consumed, d) VO2 consumed e) VCO2 expired f) respiratory quotient (a-b; two-tailed t-test, c-f; one-way ANOVA). Biochemical and ELISA studies of plasma, obtained after a 4h fast, from *Dnmt3a*$^{+/+}$ and *Dnmt3a*$^{R878H/+}$ mice fed normal chow for triglycerides by g) age and h) sex; cholesterol by i) age and j) sex; glucose by k) age and l) sex; free fatty acids (FFA) m) age and n) sex; o) leptin by sex. P-values for each comparison (via ANOVA) are indicated. Individual data points are presented and mean +/- SEM are shown. For *a-f* n=8 biologically independent replicates /genotype. For *g-o* n=10 biologically independent replicates /genotype. cm; centimeter, sec; second, VO2; oxygen consumption, VCO2, carbon dioxide output, ml; milliliter, h; hour, kg; kilogram, mg/dL; milligrams/deciliter, FFA: free fatty acid, mM; millimolar, pg; picograms.

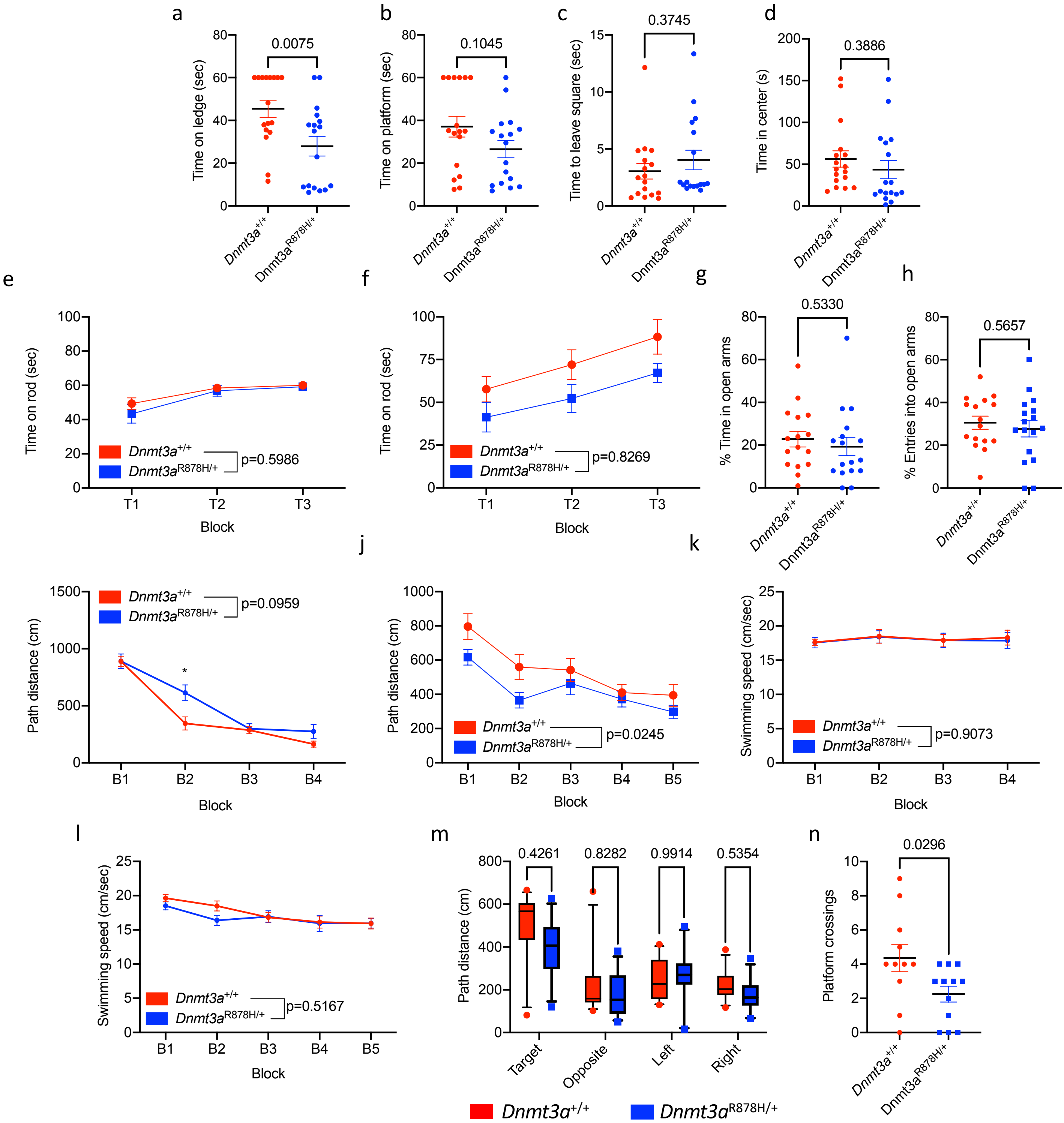

Supplementary Figure 4

Supplementary Figure 4. Evaluation of behavioral alterations in germline Dnmt3a$^{R878H/+}$ mice. a-e) Comparison of *Dnmt3a*$^{R878H/+}$ and *Dnmt3a*$^{+/+}$ mice across sensorimotor assays shows subtle alterations in a) walking initiation or latency to fall off. b) ledge (p = 0.0075, n = 17,17) or c) platform. d) Total time spent in the center zone of field during 1-hour open-field testing. e-f) Motor coordination of *Dnmt3a*$^{R878H/+}$ compared to *Dnmt3a*$^{+/+}$ littermate mice in e) continuous or f) accelerating rotarod (n = 16,17). g) Percentage of time in the open arms during elevated plus maze test (n = 16,17). h) Percentage of entries into open arms during elevated plus maze test (n = 16,17). i-j) Path distance to escape platform in Morris water maze in i) cued trials and j) place trials (p = 0.0245, genotype effect $F_{(1,21)} = 5.869$, n = 11,12). k-l) Swim speed during Morris water maze in k) cued trials and l) place trials (n = 11,12). m) Time spent in the target and other quadrants of a Morris water maze. The box extends from 25th-75th percentile, line indicate median and whiskers show 10th-90th percentile. n) Platform crossings in the probe trial (p = 0.0296, n = 11,12). Line and bar graphs indicate mean and SEM for each value tested. P-values in a-d, g, h, and n determined by two-tailed t-test. P-values in e, f, i, j, k, l and m determined by two-way repeated-measures ANOVA with Šídák's multiple comparison test. sec; seconds, cm; centimeter.

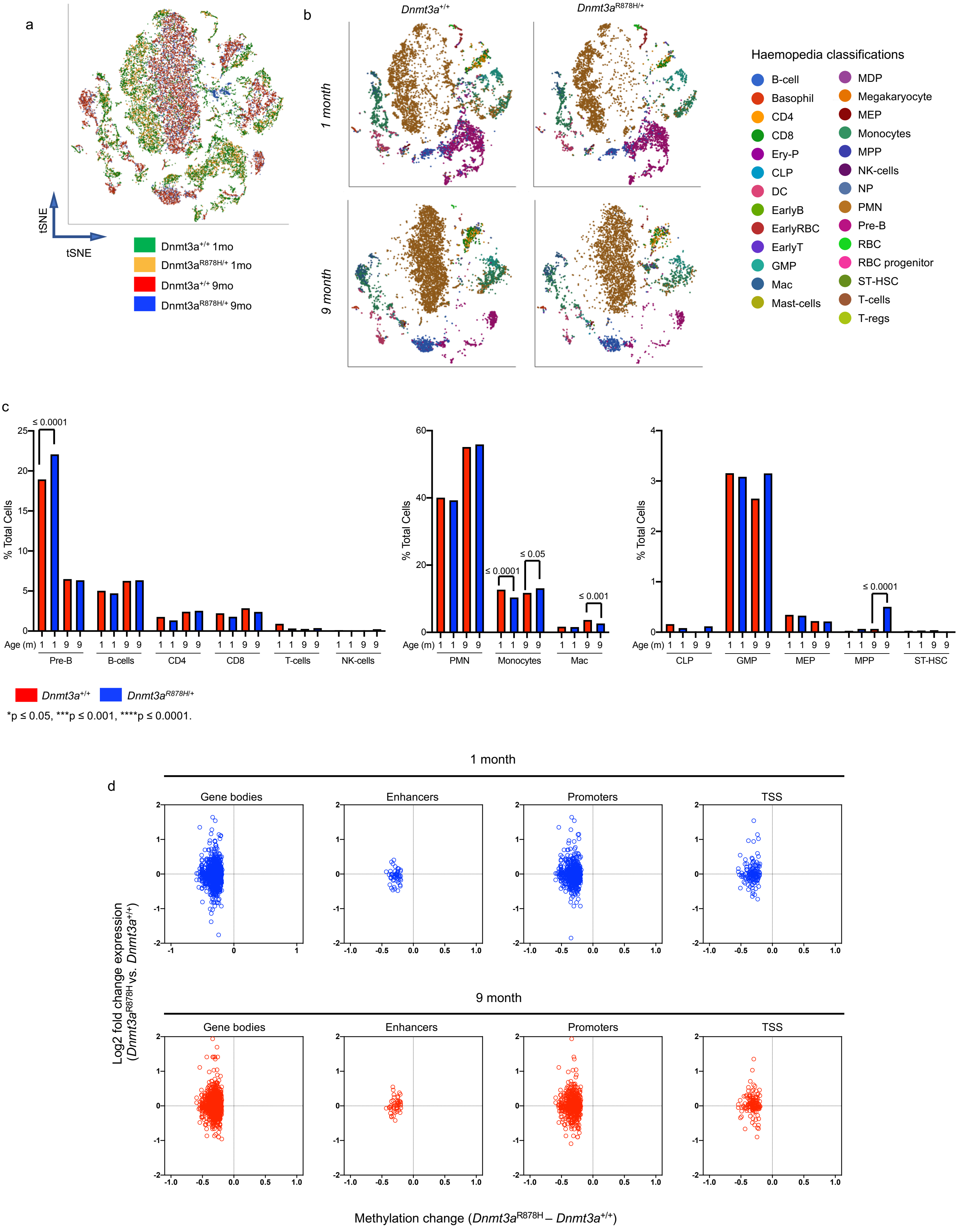

Supplementary Figure 5

Supplementary Figure 5. Germline Dnmt3a$^{R878H/+}$ mice have minimally perturbed hematopoiesis by scRNAseq. a) t-SNE projections showing overlap of all four mouse bone marrow samples used in the analysis; *Dnmt3a*$^{+/+}$ (n=2; 1 and 9 months of age) and *Dnmt3a*$^{R878H/+}$ (n=2; 1 and 9 months of age). b) individual tSNE projections of scRNA-seq data from whole bone marrow samples from *Dnmt3a*$^{+/+}$ and *Dnmt3a*$^{R878H/+}$ mice, showing known populations defined using the Haemopedia database. c) Percentage of cells in Haemopedia defined lymphoid (*left*), myeloid (*middle*) and Hematopoietic Progenitors and Stem Cell (*right*) populations identified in *b.* P-values of ratios were defined by Fisher's Exact test. d) Expression vs. methylation difference plots for all expressed genes between *Dnmt3a*$^{+/+}$ and *Dnmt3a*^{R878H} mice at 1 month (*blue, upper panels*) or 9 months (*red, lower panels*), that are associated with a DMR. Functional locations of DMRs are defined, and plotted separately (gene bodies, enhancers, promoters, and Transcription Start Sites/TSS). mo; months.

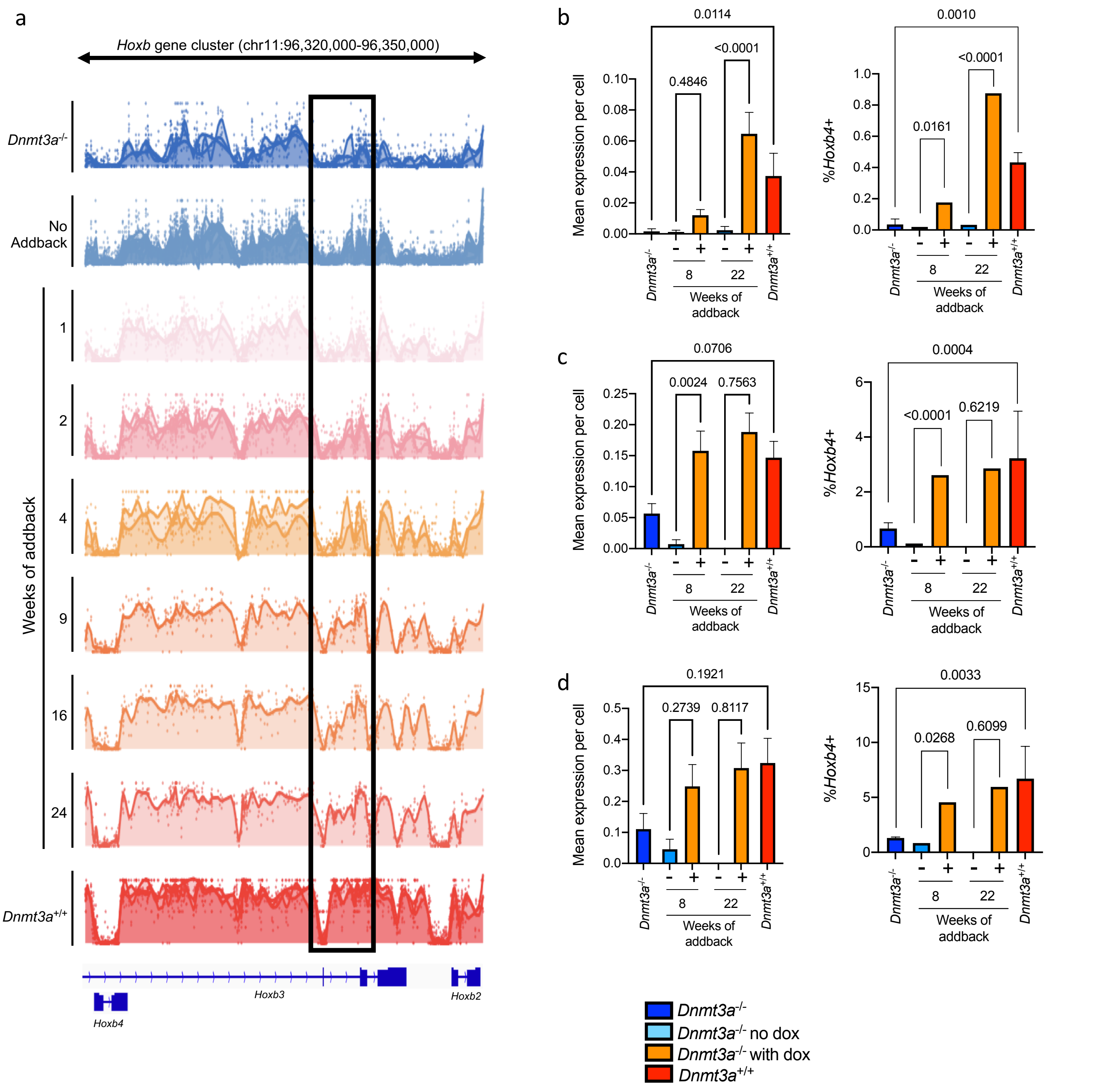

Supplementary Figure 6

Supplementary Figure 6. Remethylation of Dnmt3a-dependent DMRs is associated with increased Hoxb4 expression. a) Methylation values for a region in the *Hoxb* gene cluster in the bone marrow cells of mice harvested various times after *DNMT3A* addback. The methylation phenotype of *Dnmt3a*$^{-/-}$ bone marrow cells is shown in the top row (*blue*), vs. *Dnmt3a*$^{+/+}$ bone marrow in the bottom row (*blue*). Methylation values for *Dnmt3a*$^{-/-}$ samples re-expressing WT DNMT3A for 0 weeks (“no addback”), 1, 2, 4, 9, 16, or 24 weeks are shown in the intervening rows. A prominent remethylated DMR is highlighted in the box. Panels b, c, and d) Expression levels of *Hoxb4* per cell (left) and fractions of *Hoxb4* expressing cells (right), determined using scRNA-seq for b) PMNs, c) monocytes and d) GMPs. Expression values for *Dnmt3a*$^{-/-}$ (n=2), *Dnmt3a*$^{+/+}$ (n=2) and *Dnmt3a* null-3A addback mice treated for 8 (n=1 per treatment) or 22 (n=1 per treatment) weeks with (+) or without (-) doxycycline, which induced DNMT3A expression (“weeks of addback”), are shown. P-values defined by unpaired t-test are shown. Data are presented as mean values +/- SEM.

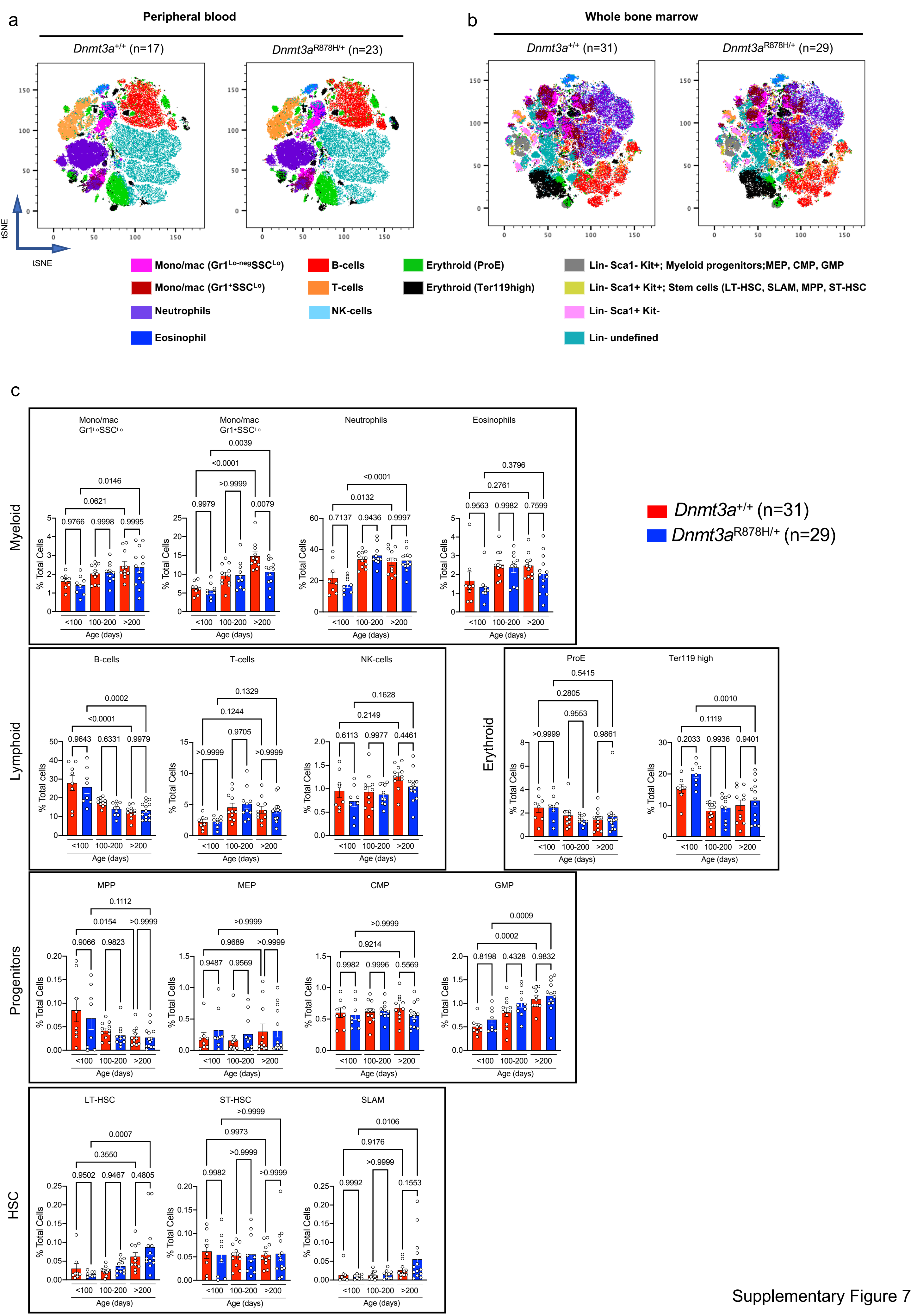

Supplementary Figure 7

Supplementary Figure 7. Germline Dnmt3a$^{R878H/+}$ mice have minimally perturbed hematopoiesis by flow cytometry. a) t-SNE projections of flow-cytometry data from peripheral blood obtained from primary *Dnmt3a*$^{+/+}$ (left panel; n=17) and *Dnmt3a*$^{R878H/+}$ (right panel; n=23) mice showing known hematopoietic populations based on defined cell surface markers. b) The t-SNE projections of flow-cytometry data from whole bone marrow obtained from primary *Dnmt3a*$^{+/+}$ (left panel; n=31) and *Dnmt3a*$^{R878H/+}$ (right panel; n=29) mice showing known hematopoietic populations based on well-defined cell surface markers. c) Quantification of population fractions associated with the flow-cytometry data shown in *b*; p-values were defined by 2-way ANOVA (n=31,29 biologically independent samples). Data are presented as mean values + SEM.

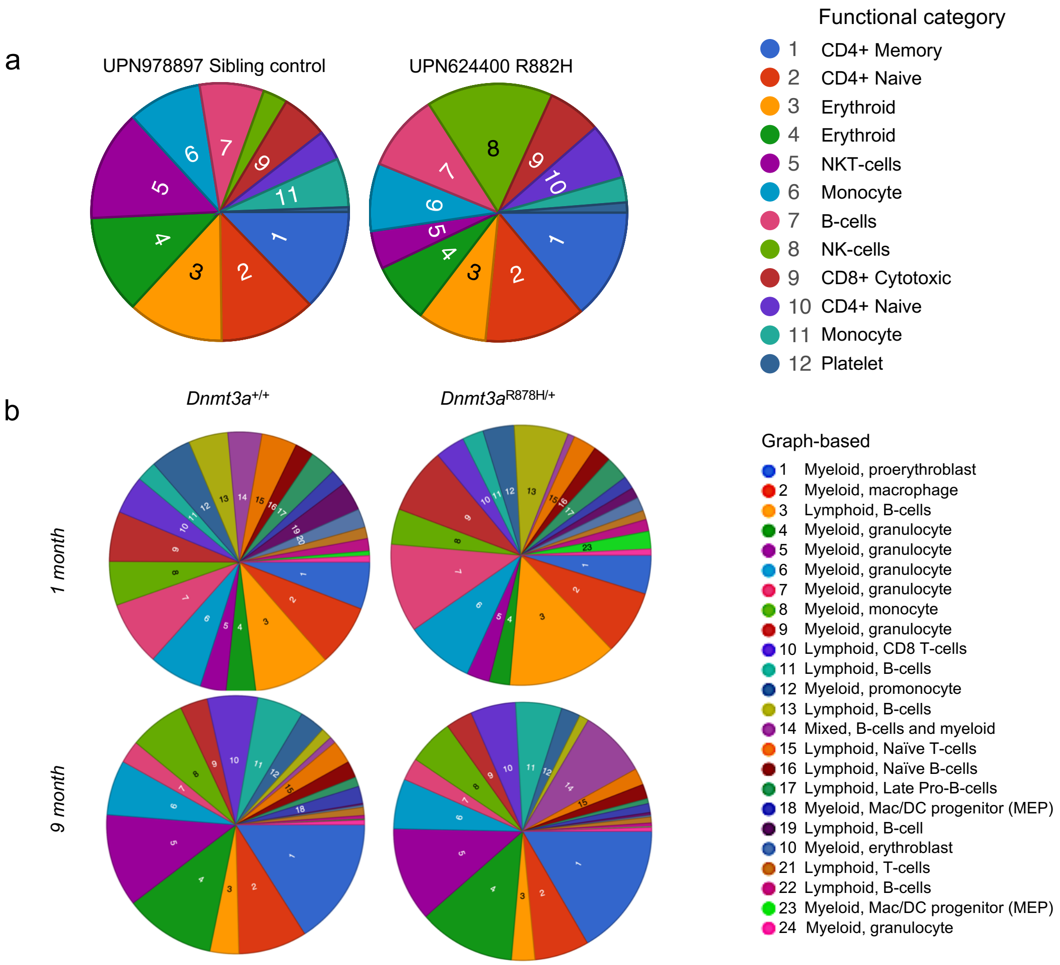

Supplementary Figure 8. a) Percentage contribution of each graph-based cluster associated with human scRNA-seq data shown in figure 2. b) Percentage contribution of each graph-based cluster associated with mouse scRNA-seq data shown in figure 6.

a

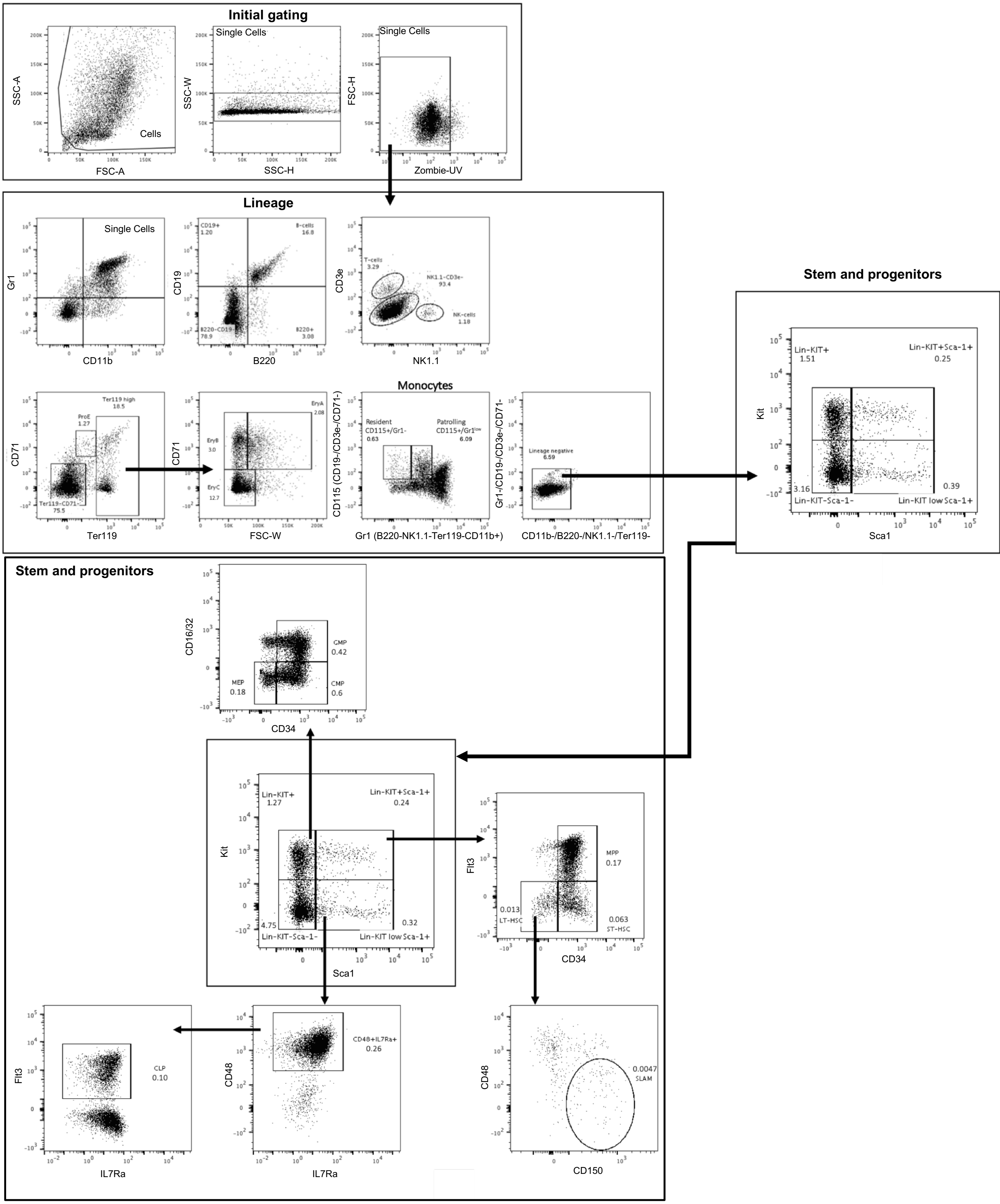

Initial gating
Cells
Single Cells
Single Cells
SSC-A
FSC-A
SSC-W
SSC-H
FSC-H
Zombie-UV
Lineage
Single Cells
Gr1
CD11b
CD19+
1.20
B-cells
16.8
B220-CD19-
78.9
B220+
3.08
CD19
B220
T-cells
3.29
NK1.1-CD3e-
93.4
NK-cells
1.18
CD3e
NK1.1
Ter119 high
18.5
ProE
1.27
Ter119-CD71-
75.5
CD71
Ter119
EryA
2.08
EryB
3.0
EryC
12.7
CD71
FSC-W
Monocytes
Resident
CD115+/Gr1-
0.63
Patrolling
CD115+/Gr1low
6.09
CD115 (CD19-/CD3e-/CD71-)
Gr1 (B220-NK1.1-Ter119-CD11b+)
Lineage negative
6.59
Gr1-/CD19-/CD3e-/CD71-
CD11b-/B220-/NK1.1-/Ter119-
Stem and progenitors
Lin-KIT+
1.51
Lin-KIT+Sca-1+
0.25
3.16
0.39
Lin-KIT-Sca-1-
Lin-KIT low Sca-1+
Kit
Sca1
Stem and progenitors
GMP
0.42
MEP
0.18
CMP
0.6
CD16/32
CD34
Lin-KIT+
1.27
Lin-KIT+Sca-1+
0.24
4.75
0.32
Lin-KIT-Sca-1-
Lin-KIT low Sca-1+
Kit
Sca1
MPP
0.17
0.013
LT-HSC
0.063
ST-HSC
Flt3
CD34
CLP
0.10
Flt3
IL7Ra
CD48+IL7Ra+
0.26
CD48
IL7Ra
0.0047
SLAM
CD48
CD150

b

Healthy Donor PBMC Control

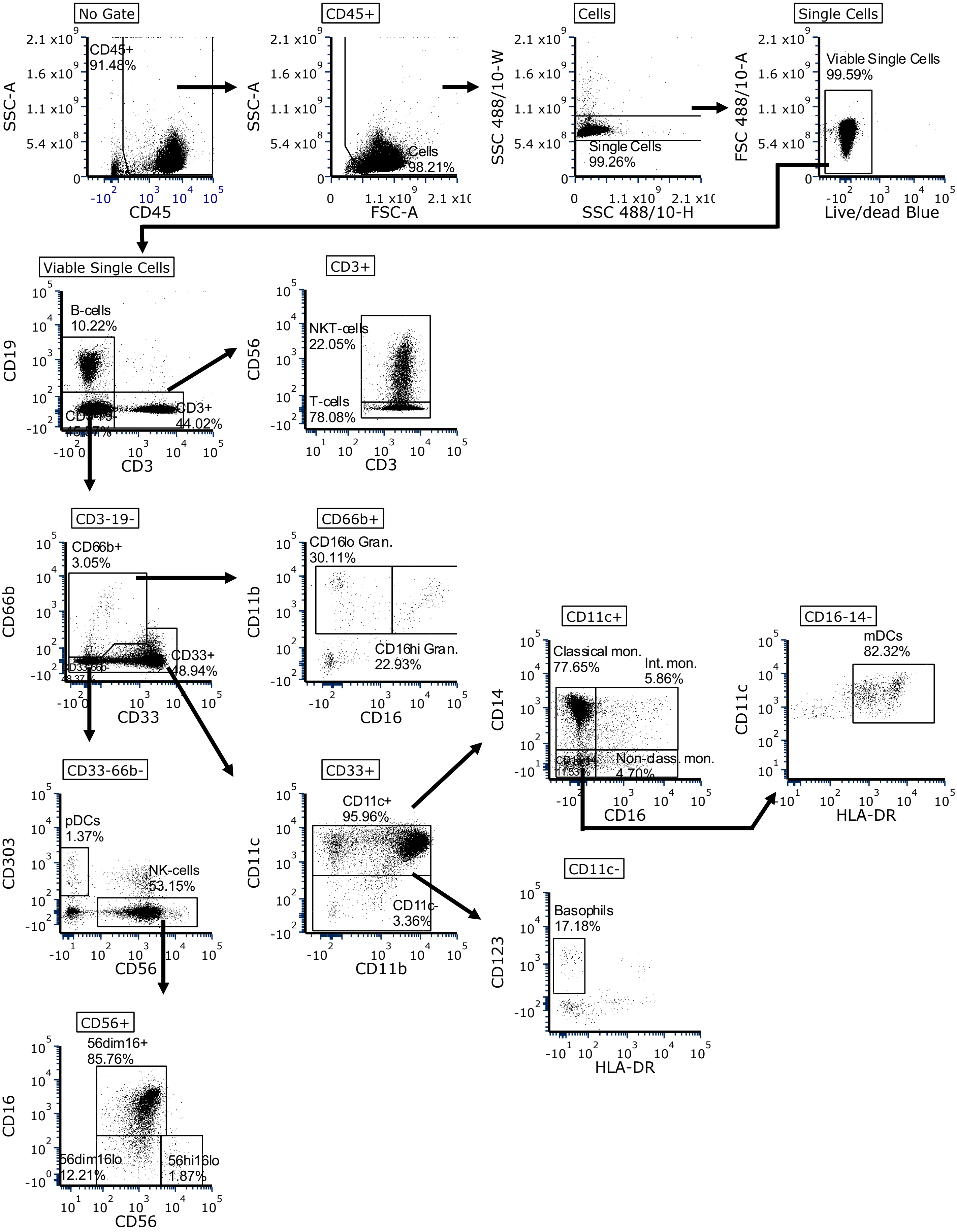

Supplementary figure 9. Gating strategy for a) human and b) mouse flow cytometric data.
