# Supplementary material for: Functional and epigenetic phenotypes of humans and mice with DNMT3A Overgrowth Syndrome
Source: Nat Commun. 2021 Jul 27;12:4549. doi: 10.1038/s41467-021-24800-7 (PMC8316576; doi:10.1038/s41467-021-24800-7)
Supplement: Supplementary file 3 — Description of Additional Supplementary Files [file 41467_2021_24800_MOESM3_ESM.pdf]

Description of Additional Supplementary Files

File Name: Supplementary Data 1
Description: **Differentially methylated regions (DMRs) identified in peripheral blood using WGBS data from DNMT3A Overgrowth Syndrome (DOS) patients with R882**
Genomic coordinates for each DMR are listed in columns A-D, the functional regions in which the DMR falls are shown in F-L, and mean methylation values for DMRs in individual samples are shown in columns N-AM. Size of DMR is in base pairs.

File Name: Supplementary Data 2
Description: **Differentially methylated regions (DMRs) identified in peripheral blood samples using WGBS data from DOS patients with non-R882 mutations.**
Genomic coordinates for each DMR are listed in columns A-D, the functional regions in which the DMR falls are shown in F-L, and mean methylation values for DMRs in individual samples are shown in columns N-AM. Size of DMR is in base pairs.

File Name: Supplementary Data 3
Description: **Differentially expressed genes (DEGs) identified using single cell RNA-seq data from the peripheral blood of a DOS patient with an R882H mutation, vs. his unaffected brother.**
DEGs were identified using scRNA-seq data from individual graph-based clusters from *DNMT3A*$^{+/+}$ and *DNMT3A*$^{R882H/+}$ peripheral blood samples. Each graph-based cluster is shown as an individual tab, as indicated. Column A: feature ID, B: p-value of expression for *DNMT3A*^{R882} vs. *DNMT3A*$^{+/+}$, C: false discovery rate (FDR), D: Ratio of expression (*DNMT3A*^{R882}/*DNMT3A*$^{+/+}$), E: Fold change of expression (highest value/lowest value, *DNMT3A*^{R882} used as numerator for directionality of change), F-G: LS means for *DNMT3A*^{R882} and *DNMT3A*$^{+/+}$ respectively.

File Name: Supplementary Data 4
Description: **Differentially expressed genes (DEGs) identified using bulk RNA-seq data from the peripheral blood from two DOS patients with R882 mutations.**
Differentially expressed genes from two DOS patients were identified by comparing to four healthy donors (*DNMT3A*$^{+/+}$); differentially expressed gene data is shown in individual tabs for each DOS patient (UPN 154605 and UPN 624400), as indicated. Column A: ensembl ID, B: chromosome, C: total read counts, D: p-value of expression for *DNMT3A*^{R882} vs. *DNMT3A*$^{+/+}$, E: Fold change of expression (highest value/lowest value, *DNMT3A*^{R882} used as numerator for directionality of change), F: log2 of Fold Change (from values in column E).

File Name: Supplementary Data 5
Description: **Differentially methylated regions (DMRs) in bone marrow cells, identified by comparing WGBS data from *Dnmt3a*$^{+/+}$ vs. germline *Dnmt3a*$^{R878H/+}$ mice.**
Genomic coordinates for each DMR are listed in columns A-D, the functional regions in which the DMR falls are shown in F-L, and mean methylation values for DMRs in individual samples are shown in columns N-AC. *Dnmt3a*$^{+/-}$ and *Dnmt3a*$^{-/-}$ values for the same DMRs are shown in AD-AG and AH-AK, respectively. Size of DMR is in base pairs.

File Name: Supplementary Data 6

Description: **Differentially methylated regions (DMRs) in bone marrow cells, identified by comparing WGBS data from *Dnmt3a*$^{+/+}$ vs. germline *Dnmt3a*$^{-/-}$ mice.**

Genomic coordinates for each DMR are listed in columns A-D, the functional regions in which the DMR falls are shown in F-L, and mean methylation values for DMRs in individual samples are shown in columns N-AA. *Dnmt3a*$^{R878H/+}$ and *Dnmt3a*$^{+/-}$ values for the same DMRs are shown in AB-AG and AH-AK, respectively. Size of DMR is in base pairs.

File Name: Supplementary Data 7

Description: **Differentially methylated regions (DMRs) in bone marrow cells, identified by comparing WGBS data from *Dnmt3a*$^{+/+}$ vs. germline *Dnmt3a*$^{+/-}$ mice.**

Genomic coordinates for each DMR are listed in columns A-D, the functional regions in which the DMR falls are shown in F-L, and mean methylation values for DMRs in individual samples are shown in columns N-AA. *Dnmt3a*$^{R878H/+}$ and *Dnmt3a*$^{-/-}$ values for the same DMRs are shown in AB-AG and AH-AK, respectively. Size of DMR is in base pairs.

File Name: Supplementary Data 8

Description: **Differentially expressed genes (DEGs) identified by comparing scRNA-seq data from the bone marrow of *Dnmt3a*$^{+/+}$ vs. germline *Dnmt3a*$^{R878H/+}$ mice.**

DEGs identified in individual graph-based clusters by comparing bone marrow samples from 1-month and 9-month-old *Dnmt3a*$^{+/+}$ vs. germline *Dnmt3a*$^{R878H/+}$ mice. Each tab represents expression data of DEGs identified in individual graph-based clusters in the 1-month old or 9-month old sample pair, as indicated. Column A: Gene Symbol, B: chromosome, C: p-value of expression for *Dnmt3a*$^{R878H/+}$ vs. *Dnmt3a*$^{+/+}$, D: false discovery rate (FDR), E: Ratio of expression (*Dnmt3a*$^{R878H/+}$ / *Dnmt3a*$^{+/+}$), F: log2 of ratio (column E), G: Fold change of expression (highest value/lowest value, *Dnmt3a*$^{R878H/+}$ used as numerator for directionality of change), F-G: LS mean values for *Dnmt3a*$^{R878H/+}$ and *Dnmt3a*$^{+/+}$ respectively.
