# Supplementary material for: Functional and epigenetic phenotypes of humans and mice with DNMT3A Overgrowth Syndrome
Source: Nat Commun. 2021 Jul 27;12:4549. doi: 10.1038/s41467-021-24800-7 (PMC8316576; doi:10.1038/s41467-021-24800-7)
Supplement: Supplementary file 12 — Reporting Summary [file 41467_2021_24800_MOESM12_ESM.pdf]

nature research

Corresponding author(s): Timothy J Ley

Last updated by author(s): Jul 1, 2021

Reporting Summary

Nature Research wishes to improve the reproducibility of the work that we publish. This form provides structure for consistency and transparency in reporting. For further information on Nature Research policies, see our Editorial Policies and the Editorial Policy Checklist.

Statistics

For all statistical analyses, confirm that the following items are present in the figure legend, table legend, main text, or Methods section.

n/a	Confirmed	
☐	☒	The exact sample size (*n*) for each experimental group/condition, given as a discrete number and unit of measurement
☐	☒	A statement on whether measurements were taken from distinct samples or whether the same sample was measured repeatedly
☐	☒	The statistical test(s) used AND whether they are one- or two-sided<br>*Only common tests should be described solely by name; describe more complex techniques in the Methods section.*
☐	☒	A description of all covariates tested
☐	☒	A description of any assumptions or corrections, such as tests of normality and adjustment for multiple comparisons
☐	☒	A full description of the statistical parameters including central tendency (e.g. means) or other basic estimates (e.g. regression coefficient) AND variation (e.g. standard deviation) or associated estimates of uncertainty (e.g. confidence intervals)
☐	☒	For null hypothesis testing, the test statistic (e.g. *F*, *t*, *r*) with confidence intervals, effect sizes, degrees of freedom and *P* value noted<br>*Give P values as exact values whenever suitable.*
☐	☒	For Bayesian analysis, information on the choice of priors and Markov chain Monte Carlo settings
☐	☒	For hierarchical and complex designs, identification of the appropriate level for tests and full reporting of outcomes
☐	☒	Estimates of effect sizes (e.g. Cohen's *d*, Pearson's *r*), indicating how they were calculated

Our web collection on statistics for biologists contains articles on many of the points above.

Software and code

Policy information about availability of computer code

Data collection	Flow cytometry data was acquired with Everest (3.0.59.0)
Data analysis	All software used for primary genomic analyses are described in the Methods section, including links to the github repo in which they are available, with code and docker containers necessary to reproduce the analyses. Software for DMR calling (metilene v0.2.8) is likewise available openly at https://www.bioinf.uni-leipzig.de/Software/metilene/Downloads/. Other statistical tests and comparisons were performed using either R version 4.0.2, or via Partek Flow software (10.0.21.0411), or via GraphPad Prism (9.1.2). Flow cytometry analysis was performed using FlowJo (10.7.1).

For manuscripts utilizing custom algorithms or software that are central to the research but not yet described in published literature, software must be made available to editors and reviewers. We strongly encourage code deposition in a community repository (e.g. GitHub). See the Nature Research guidelines for submitting code & software for further information.

Data

Policy information about availability of data

All manuscripts must include a data availability statement. This statement should provide the following information, where applicable:

- Accession codes, unique identifiers, or web links for publicly available datasets
- A list of figures that have associated raw data
- A description of any restrictions on data availability

Sequencing data for all mouse datasets were deposited to the NCBI, (https://www.ncbi.nlm.nih.gov/bioproject/PRJNA722276).

Sequencing data for all human datasets were deposited to dbGaP study # phs000159.v11.p5, https://www.ncbi.nlm.nih.gov/projects/gap/cgi-bin/study.cgi?

study_id=phs000159.v11.p5

Field-specific reporting

Please select the one below that is the best fit for your research. If you are not sure, read the appropriate sections before making your selection.

☒ Life sciences ☐ Behavioural & social sciences ☐ Ecological, evolutionary & environmental sciences

For a reference copy of the document with all sections, see nature.com/documents/nr-reporting-summary-flat.pdf

Life sciences study design

All studies must disclose on these points even when the disclosure is negative.

Sample size	Human: Sample size was determined by availability and willingness of families of affected individuals to participate. Mouse: A minimum of 3 independent biological replicates is the industry standard for WGBS data analysis and we exceeded this number for all genotypes utilised in the study.
Data exclusions	All data was included.
Replication	We utilized as many biological replicates for the human data as possible based on families willing to participate. All data included were based on biological replicates, not technical replicates, unless specifically indicated. We also used both male and female samples with a range of ages in both human and mouse data that was assessed for contribution to phenotypes with covariate analysis and is described in the manuscript.
Randomization	No randomisation was performed as this was not a clinical trial involving treatments or interventions.
Blinding	No blinding was performed as this was not a clinical trial involving treatments or interventions.

Reporting for specific materials, systems and methods

We require information from authors about some types of materials, experimental systems and methods used in many studies. Here, indicate whether each material, system or method listed is relevant to your study. If you are not sure if a list item applies to your research, read the appropriate section before selecting a response.

Materials & experimental systems

n/a	Involved in the study	
☐	☒	Antibodies
☒	☐	Eukaryotic cell lines
☒	☐	Palaeontology and archaeology
☐	☒	Animals and other organisms
☐	☒	Human research participants
☒	☐	Clinical data
☒	☐	Dual use research of concern

Methods

n/a	Involved in the study	
☒	☐	ChIP-seq
☐	☒	Flow cytometry
☒	☐	MRI-based neuroimaging

Antibodies

Antibodies used	All antibodies used were all commercially acquired. Mouse: CD11b (MI/70, #612977), Gr-1 (TB6-8C5; Biolegend, #108456), Ter-119 (Ter119, #135524), CD71 (C2, #563013), B220 (RA3-6B2, #612950), CD19 (6D5, Biolegend, 115525), CD3e (145-2C11, #612771), NK1.1 (PK136, #564143), Sca-1 (D7; Biolegend, #122528), c-KIT (2B8, #562609), CD34 (RAM34, #553733), FLT3 (A2F10; Biolegend, #135306), CD150 (TC15-12F12.2; Biolegend, #115941), CD48 (HM48-1; Biolegend, #103420), Ly5.1 (A20, #110728), Ly5.2 (104, #740131). Human:CD45 (HI30, #563792), CD3 (UCHT1, #612964), CD11b (MI/70, #612801), CD16 (3G8; BioLegend, #302037), CD19 (HIB19, BioLegend, #302243), CD14 (M5E2, BioLegend, #301835), HLA-DR (G46-6, #564041), CD15 (HI98, #555401), CD56 (NCAM16.2, #340363), CD33 (WM53, #562492), CD11c (B-ly6, #561356), CD66b (G10F5, BioLegend, #305117), CD123 (32703, R&D systems, #FAB301N), CD303 (REA693, Miltenyi, #130-114-177)
Validation	Antibody titration was performed for each individual antibody and these antibodies and clones are well established in the field of hematology research. 5e6 whole bone marrow cells were used to titrate antibodies from 0.015625 to 2ul per 5e6 cells.

Animals and other organisms

Policy information about studies involving animals; ARRIVE guidelines recommended for reporting animal research

Laboratory animals	Mus musculus, C57Bl/6, both male and females used and ages from 2-52 weeks as annotated in manuscript and figures. Mice were housed in a 12h light cycle facility at 23 degrees Celsius with 50% humidity.
Wild animals	Wild animals were not used in this study
Field-collected samples	Field collected samples were not used in this study
Ethics oversight	Studies approved by the Animal Studies Committee at Washington University

Note that full information on the approval of the study protocol must also be provided in the manuscript.

Human research participants

Policy information about studies involving human research participants

Population characteristics	Human patients were male and female with a prior diagnosis of DNMT3A overgrowth syndrome defined by positive test for germline DNMT3A mutations. Patients aged from 1.7-36 years of age as annotated in manuscript and figures. Controls were defined as germline DNMT3A WT, ranged in age from 4-43 years of age and were male and female.
Recruitment	We contacted the TBRS community who advertised the study on their website (https://tbrsyndrome.org) and families interested in participating contacted us. All participants provided informed consent. No bias will be present as any family who asked to be included in the study and consented were included in the study.
Ethics oversight	Studies were approved by the Human Research Protection Office at Washington University School of Medicine following informed consent in accordance with the Declaration of Helsinki. Patient samples were collected following consent using an IRB protocol #201011766. Explicit permission for whole genome sequencing is provided in this protocol.

Note that full information on the approval of the study protocol must also be provided in the manuscript.

Flow Cytometry

Plots

Confirm that:

- [x] The axis labels state the marker and fluorochrome used (e.g. CD4-FITC).
- [x] The axis scales are clearly visible. Include numbers along axes only for bottom left plot of group (a 'group' is an analysis of identical markers).
- [x] All plots are contour plots with outliers or pseudocolor plots.
- [x] A numerical value for number of cells or percentage (with statistics) is provided.

Methodology

Sample preparation	Mouse:Peripheral blood and bone marrow from mouse femurs and tibias were utilized for flow cytometry applications. Once isolated, cells were treated with ACK red cell lysis buffer. Human: Peripheral blood from unaffected controls and DOS patients were processed to buffy coat preparations, and then ACK red cell lysis buffer was used to remove persistent red cells before flow cytometry with a 15 antibody panel was performed.
Instrument	ZE5 (Bio-rad) was used for all acquisition
Software	Acquisition was performed using Everest software and analysis with FlowJo (Tree Star) and Prism 9 (GraphPad)
Cell population abundance	No cell sorting was performed in this manuscript
Gating strategy	We used industry defined standards for population identification (including well established Weissman progenitors) and recently described in Ketkar et al 2020 PNAS from our lab. We utilise a comprehensive 21 colour flow cytometry protocol and no single gating strategy can be shown to describe all populations in full. Standard FSC/SSC gating was used to gate all WBM cells- we did not use FSC/SSC to gate individual cell types ie lymphs, mono, granulocytes.

- [x] Tick this box to confirm that a figure exemplifying the gating strategy is provided in the Supplementary Information.
